# The pharmacological bases of the antiangiogenic activity of paclitaxel

**DOI:** 10.1007/s10456-013-9334-0

**Published:** 2013-02-07

**Authors:** Guido Bocci, Antonello Di Paolo, Romano Danesi

**Affiliations:** Division of Pharmacology, Department of Clinical and Experimental Medicine, University of Pisa, Via Roma 55, 56126 Pisa, Italy

**Keywords:** Paclitaxel, Antiangiogenic drugs, Angiogenesis, Preclinical studies

## Abstract

In the mid 1990s, researchers began to investigate the antiangiogenic activity of paclitaxel as a possible additional mechanism contributing to its antineoplastic activity in vivo. In the last decade, a number of studies showed that paclitaxel has antiangiogenic activity that could be ascribed to the inhibition of either tubule formation or cell migration, and to an antiproliferative effect towards activated endothelial cells. Furthermore, paclitaxel was shown to downregulate VEGF and Ang-1 expression in tumor cells, and to increase the secretion of TSP-1 in the tumor microenvironment. Moreover, the new pharmaceutical formulations of paclitaxel (such as liposome-encapsulated paclitaxel, ABI-007, and paclitaxel entrapped in emulsifying wax nanoparticles) enhanced the in vivo antiangiogenic activity of the drug. Thus, the preclinical data of paclitaxel may be exploited to implement a novel and rational therapeutic strategy to control tumor progression in patients.

## Introduction

Paclitaxel is a cancer chemotherapeutic agent that is commonly used as first line therapy for many common malignancies, including lung, breast, ovarian and head and neck cancer. It also has high antitumor activity against some uncommon malignancies, such as angiosarcoma and Kaposi sarcoma. Paclitaxel is a member of the taxane class of drugs, and it was the first taxane to enter clinical trials and to receive FDA approval; it is a natural product obtained from the North American Pacific yew tree (*Taxus brevifolia*). Paclitaxel binds to the beta-subunit of polymerized tubulin and inhibits the dissociation rate of the tubulin subunits from the tubule. Thus, micromolar paclitaxel administration to cells results in the formation of microtubule bundles and asters, arresting cells in mitosis [1].

Besides these known pharmacodynamic properties, medical researchers have discovered a new and unexpected additional characteristic of paclitaxel, one that started a second life for this compound and that created new therapeutic approaches using this molecule: that characteristic is its antiangiogenic activity. Although the anti-vascular effects of tubulin-binding agents have been previously reviewed [2–4], the present article focuses solely on the pharmacological bases of the antiangiogenic activity of paclitaxel and provides an extensive and updated overview of the field. Table 1 summarizes the published data on the antiangiogenic effects of paclitaxel in different in vitro and in vivo experimental models.

**Table 1:** Pharmacological effects of paclitaxel on angiogenesis

Models	Pharmacological effects at cellular level	Pharmacological effects at molecular level	References
**In vitro**			
HUVEC	Strong anti-proliferative activity		[8]
HUVEC	Inhibition of cell proliferation, motility and invasiveness in a concentration-dependent manner		[6]
HMVEC-d, HUVEC	Selective inhibition of cell proliferation and induction of apoptosis at low concentrations for prolonged periods of time		[18]
Human leukemia cell lines		VEGF downregulation in vitro (even in drug resistant cells)	[9]
HUVEC	Reduction of the capillary network formation in matrigel		[20]
Rat fat pad endothelial cells	Inhibition of migration		[21]
HUVEC, rat aortic ring explants	Inhibition of cell proliferation, migration, and tube formation at one-tenth the concentration needed to achieve a similar effect on tumor cell lines		[22]
HUVEC, rat aortic ring explants	Inhibition of proliferation, differentiation (tube assay) and induction of cell death		[23]
HUVEC, HMEC-1	Initiation, without completion, of the mitochondrial apoptotic pathway leading to a slowing down of the cell cycle	Cytotoxic effects mediated by microtubule network disturbance, G2-M arrest, increase in Bax/Bcl-2 ratio, and mitochondria permeabilization	[40]
HUVEC, HMEC-1		Increase of interphase microtubule dynamics in vitro	[41]
HUVEC, HMVEC-L, HMVEC-D	Inhibition of FGF-2- and VEGF-induced cell proliferation and tube formation in matrigel	Increase of the drug cellular uptake in endothelial cells *vs.* fibroblasts and tumor cells (mechanism unknown)	[35]
HUVEC		Induction of gene and protein expression of TSP-1 at metronomic concentrations	[45]
Human ovarian cancer cell lines		Decrease of survival factors such as Ang-1 and VEGF	[47]
Rat bone marrow (BM)-derived endothelial progenitor cells (EPC) cell line (TR-BME)	Inhibition of tube formation and migration of cells at low concentrations		[34]
HUVEC	Inhibition of migration	Increase of the levels of acetylated tubulin; increase of forkhead box O3a translocation into the nucleus	[44]
**In vivo**			
Nude mice bearing murine breast cancer		VEGF downregulation	[9]
Cornea assay	Inhibition of FGF-2 and VEGF-induced neovascularization		[7]
Transgenic murine Met-1 breast cancer model	Reduced intratumor angiogenesis	VEGF downregulation	[9]
Human oral squamous cell carcinoma, lung tumor	Inhibition of tumor angiogenesis	Reduced the immunohistochemical expression of CD31, VEGF and VEGF mRNA	[11, 12]
Melanoma spontaneous metastases model	Inhibition of angiogenesis in melanoma tissue lesions	Reduction of VEGF-A expression	[25]
Rat Walker 256 breast carcinosarcoma cell xenografts	A low dose inhibits bone marrow-derived endothelial progenitor cells (EPC) accumulation at the tumor site and decrease the microvessel density		[34]
Chick chorioallantoic membrane	Inhibition of neovascularization		[5]
Matrigel pellet in mice	Inhibition of neovascularization		[6]
Chick chorioallantoic membrane	Inhibition of neovascularization at low concentrations		[26]
Rat mesentery assay	A low dose shortened the length of sprouts in VEGF-mediated angiogenesis		[27, 28]
4T1 metastatic breast cancer	Strong antiangiogenic and anti-lymphangiogenic activities of low doses		[30]
Rats bearing syngeneic prostate cancer (Dunning AT-1) not expressing TSP-1		Re-induction of TSP-1 expression in tumors	[46]
HT-29 colon cancer model; 4T1 metastatic breast cancer		Upregulation of TSP-1 expression	[29, 30]
Ovarian carcinoma xenograft model		Downregulation of VEGF-B, -D and -A; upregulation of Tie-1, Tie-2 and VEGFR-2	[49]

The first ever published evidence about a possible antiangiogenic activity of paclitaxel was a small report some 15 years ago. Dordunoo and colleagues prepared poly(epsilon-caprolactone) (PCL) microspheres containing paclitaxel, and showed this formulation to inhibit angiogenesis in the chick chorioallantoic membrane (CAM) model [5]. A year later, Belotti et al. [6] demonstrated a strong antiangiogenic activity of paclitaxel, suggesting that this property might contribute to its antineoplastic activity in vivo. The aim of that study was to investigate the effect of paclitaxel on endothelial cell functions, and on angiogenesis. In vivo, paclitaxel (20–28 mg/kg i.v.) significantly inhibited the angiogenic process in a pellet of matrigel containing tumor cell supernatant, that was injected into mice. In vitro, paclitaxel inhibited endothelial cell proliferation, motility, and invasiveness in a concentration-dependent manner [6]. Interestingly, the authors stated that the antiangiogenic activity of paclitaxel was not linked to its cytotoxicity, since inhibition of endothelial cell chemotaxis and invasiveness occurred at drug concentrations which did not affect endothelial cell proliferation [6]. At the same time, a lower dose of paclitaxel (6 mg/kg i.p.) inhibited the bFGF and VEGF-induced neovascularization of the cornea in mice by 45 and 37 %, respectively [7]. Moreover, Iwahana et al. [8] demonstrated a different chemosensitivity of human umbilical vein endothelial cells (HUVEC) and of tumor-derived endothelial cells from rat KMT-17 fibrosarcoma (TEC) when exposed to the same paclitaxel concentrations. Indeed, paclitaxel had strong anti-proliferative activity against HUVECs, but only weakly inhibited the proliferation of TECs, which expressed greater amounts of P-glycoprotein (P-gp), suggesting a drug resistant phenotype of newly formed capillaries [8].

The first evidence of the antiangiogenic activity of paclitaxel via down-regulation of vascular endothelial growth factor (VEGF) in tumors was obtained in a highly-vascularized transgenic murine Met-1 breast cancer model [9]. Paclitaxel was administered intraperitoneally, at non-cytotoxic doses of 0–6 mg/kg/day, to nude mice bearing Met-1 breast tumor. Interestingly, the intratumoral angiogenesis, measured by microvessel tortuosity and microvessel density, was significantly reduced by paclitaxel treatment in a dose-dependent manner. Moreover, paclitaxel also suppressed expression of VEGF in Met-1 tumors transplanted in nude mice [9]. Interestingly, paclitaxel monochemotherapy (175 mg/m^2^ i.v. every 21 days for three cycles) significantly decreased VEGF plasma levels in metastatic breast cancer patients with partial response or stable disease, whereas no decline was observed in patients with progressive disease [10]. Paclitaxel also showed an inhibitory effect on tumor angiogenesis in a lung tumor xenograft [11], and in transplanted human oral squamous cell carcinoma [12] models, reducing the immunohistochemical expression of CD31 (an endothelial marker), VEGF, and VEGF mRNA.

However, no unanimous consensus was expressed on the antiangiogenic activity of paclitaxel in vivo. A paclitaxel dosage of 6.25 mg/kg s.c., given five times/week, slowed tumor growth in a CWR22R androgen-independent xenograft model of prostate cancer, without any antiangiogenic effects [13]. Similarly, vascular density was not altered by paclitaxel treatment in an orthotopic pancreatic tumor model [14].

## The metronomic concept and paclitaxel

**Fig. 1:**
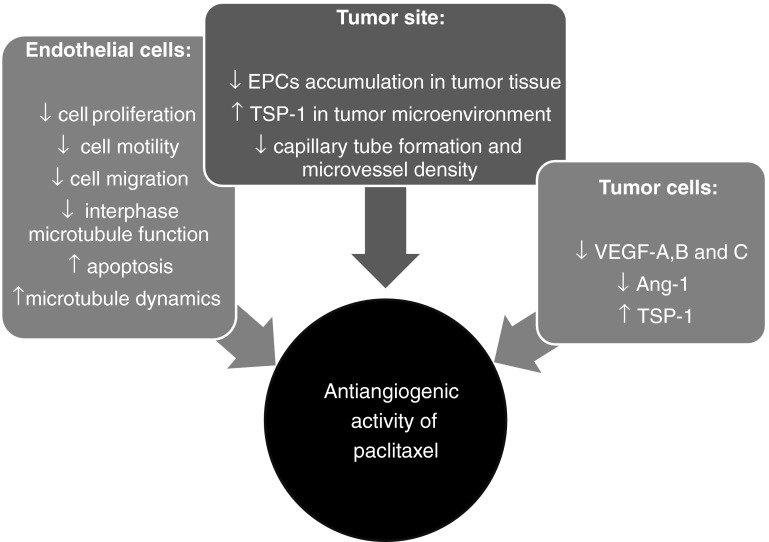
Antiangiogenic activity of paclitaxel. *EPC* endothelial progenitor cells, *TSP-1* thrombospondin-1, *Ang-1* angiopoetin-1, *VEGF* vascular endothelial growth factor

Kerbel et al. [15], at the beginning of this century, coined the term “accidental angiogenesis inhibitors” to describe a number of drugs that were tested in clinical trials as possible angiogenesis inhibitors, although they were not originally developed to target tumor angiogenesis. This list of “accidental” angiogenesis inhibitors included also established agents such as conventional cytotoxic chemotherapeutic drugs. The antivascular side-effects of chemotherapy were found to be most evident by administering such drugs continuously, or on a frequent (e.g. weekly or even daily) basis, at concentrations well below their maximum tolerated dose (MTD). This continuous low-dose schedule was defined as metronomic chemotherapy [16]. Such a frequent administration in vitro and in vivo of low doses of chemotherapeutic drugs affects the tumor endothelium and inhibits tumor angiogenesis, with minimal concomitant side effects [17–19]. In particular, low concentrations of paclitaxel showed only weak effects on in vitro activated endothelial cells using a short-term exposure (24 h) protocol, whereas a striking toxicity toward vascular endothelial cells was observed by a long-term exposure protocol (144 h) that used very low paclitaxel concentrations [18]. Moreover, a potent differential effect was found at the same paclitaxel concentrations if fibroblasts, and drug-sensitive or multidrug-resistant breast cancer cell lines were used. Indeed, a growth inhibitory effect, as well as induction of apoptosis, was observed with IC_50_ values in the range of 25–143 pM for paclitaxel only in the endothelial cells, suggesting a selective antiangiogenic therapeutic window for this taxane [18]. At the same time, Hayot et al. [20] demonstrated that paclitaxel, vincristine and vindesine, at non-cytotoxic concentrations, similarly reduced the capillary network formation by HUVEC cells cultured on Matrigel. Furthermore, paclitaxel was also tested in a chemokinetic migration assay using rat fat pad endothelial cells (RFPECs), showing IC_50_ values that were approximately 10^−9^ M [21]. The different sensitivity of activated endothelial cells to paclitaxel was also confirmed by other studies. Dicker et al. [22] found that paclitaxel inhibited human endothelial cell proliferation, migration, and tube formation (differentiation) at one-tenth of the concentration needed to achieve a similar effect on tumor cell lines (Fig. 1). Moreover, similar results were obtained on the proliferation, migration, and differentiation, of cultured human umbilical vein endothelial cells and in the capillary sprouting of rat aortic ring explants, demonstrating that endothelial cells are 10–100-fold more sensitive to paclitaxel than are tumor cells [22]. Additionally, it was demonstrated that angiogenesis could be blocked by paclitaxel, and that this effect was primarily due to inhibition of proliferation and differentiation (as measured by Matrigel assay) and by the induction of cell death [23]. The selectivity of paclitaxel inhibition of cell proliferation is also species specific. Indeed, the inhibition of proliferation of human endothelial cells by paclitaxel was observed at ultra low concentrations (0.1–100 pM), whereas 10^4^ to 10^5^-fold higher concentrations were necessary to impact human non-endothelial cells, and nonetheless mouse endothelial cells were insensitive to such ultra low (pM) concentrations of paclitaxel [24].

The antiangiogenic activity of paclitaxel at lower doses was also demonstrated in in vivo models of neovascularization, such as the chick embryo chorioallantoic membrane (CAM) [25], where paclitaxel administered at low concentrations (4, 8, and 12 nM) produced a significant dose-dependent antiangiogenic activity [26]. Likewise, using the rat mesentery assay, the effect of paclitaxel at a low, atoxic dose, significantly shortened the length of sprouts at the edge of the expanding network in VEGF165-mediated angiogenesis [27]. Moreover, metronomic paclitaxel significantly suppressed VEGF-A-mediated angiogenesis in the rat mesentery to an extent that closely mirrored the significant increase in prostate AT1 tumor necrosis, and the concomitant decrease in tumor growth rate [28]. Paclitaxel metronomic chemotherapy, in colon cancer xenografts, showed stronger antiangiogenic activity than did paclitaxel administered at the maximum tolerable dose (MTD). In contrast, the MTD chemotherapy induced more apoptotic cells than did the metronomic dosage [29]. More interestingly, Jiang et al. [30] investigated the use of low-dose metronomic (LDM) paclitaxel, as a single agent, in a highly metastatic mouse model of 4T1 breast cancers, and compared it with the maximum tolerable dose (MTD) therapy. LDM therapy displayed a stronger anti-tumor activity in suppressing primary and metastatic breast tumors, with less side effects and stronger anti-angiogenic and anti-lymphangiogenic activities, than MTD therapy. On the other hand, MTD therapy showed stronger pro-apoptotic and anti-proliferative activities in situ. Based on these results, the authors suggested the use of metronomic paclitaxel therapy to treat advanced breast cancer [30]. Metronomic paclitaxel has been successfully applied in preclinical models of metastatic tumors. In one protocol paclitaxel was injected i.p. at 5 mg/kg per day for 3 weeks to treat preclinical spontaneous melanoma metastasis. This protocol resulted in a relative decrease in spontaneous metastases to the lungs, inhibiting angiogenesis in melanoma tissue lesions and reducing the expression of VEGF-A [31]. Moreover, the weekly paclitaxel administration was also effective in inhibiting angiogenesis, tumor growth, and bone marrow hematopoiesis in a lung cancer model [32]. Indeed, the tumor growth rate was reduced by 50 % and the intratumoral microvasculature was inhibited by 70 %, with a concomitant low degree of leukopenia [32]. This important inhibition of tumor neovascularization by paclitaxel was also confirmed in a model of human breast cancer specimens grown in a fibrin clot. Interestingly, paclitaxel significantly reduced the angiogenic sprouting and the formation of new microvessels if compared to vehicle-treated controls [33]. Furthermore, low dose (non-cytotoxic) paclitaxel inhibited bone marrow-derived endothelial progenitor cells accumulation at the tumor site in tumor-bearing rats, and caused a decrease in microvessel density [34] (Fig. 1).

A possible explanation for preclinical reports describing the endothelial cell’s in vitro sensitivity to paclitaxel, the in vivo antiangiogenic effects of the drug, and clinical reports of the relative efficacy of low weekly doses of paclitaxel in patients refractory to standard (every 21 days) doses of this agent came from the study by Merchan et al. [35]. The authors observed that human endothelial cells can accumulate paclitaxel at more than 5 times greater levels than do normal human fibroblasts and several human cancer cell lines. However, the mechanism by which the endothelial cells accumulate paclitaxel remains to be elucidated. Two possibilities have been suggested: (1) the increased transport of the drug into the cells or, (2) the decrease in the efflux of the drug [35].

The effects of paclitaxel on the inflammatory process (that is a characteristic of cancer progression) and in the tumor immune escape process are still quite controversial. The induction of pro-inflammatory genes and proteins by paclitaxel, especially through the lipopolysaccharide (LPS) signaling pathway, have been reviewed by Fitzpatrick and Wheeler [36]. Moreover, Olsen [37] have described that taxanes (including paclitaxel) promote transcription of the cyclo-oxygenase (COX)-2 gene and the stabilization of the COX-2 messenger RNA. This pharmacodynamic property provided the rationale for the use of COX-2 inhibitors in combination with taxanes, and preclinical studies have shown enhanced anticancer activity from the addition of COX-2 inhibitors to taxane treatment [37]. However, more recent studies suggest that paclitaxel could modulate tissue factor (TF), as is expressed by tumour-associated endothelial and inflammatory cells (as well as by cancer cells themselves), in human mononuclear cells, in HUVECs, and in the metastatic breast carcinoma cell line MDA-MB-231 [38]. Indeed, paclitaxel significantly reduced the strong TF activity expressed by MDA-MB-231, and the TF levels in mononuclear cells and HUVECs even after the induction by LPS, TNF-α and IL-1β. Interestingly, Napoleone et al. [38] also tested whether paclitaxel could influence IL-6 and IL-1β release from the MDA-MB-231, HUVEC and mononuclear cells, since paclitaxel was shown to induce the expression of inflammatory genes in monocytes and in tumour cells. The authors demonstrated that neither the constitutive expression of IL-6 and IL-1β in MDA-MB-231, nor the basal and LPS-induced release from mononuclear cells or from HUVECs, was affected by paclitaxel administration. Recently, another important finding from Chen et al. [39] underlines the importance of the effects of paclitaxel on immunity. These authors showed that the antitumor activity of metronomic low doses of paclitaxel depends on both its capacity to deplete regulatory T cells, and on its inhibition of tumor angiogenesis. Moreover, the combination of metronomic paclitaxel plus an antigen-specific DNA vaccine induced a more potent antigen-specific immune responses and antitumor effects compared to the vaccine alone [39]. In conclusion, the effect of paclitaxel on inflammation and immunity seems to be more complex than previously hypothesized and surely merits further investigations for its relevant role in the antitumor effect of this taxane.

## Molecular mechanisms of paclitaxel antiangiogenic activity

**Fig. 2:**
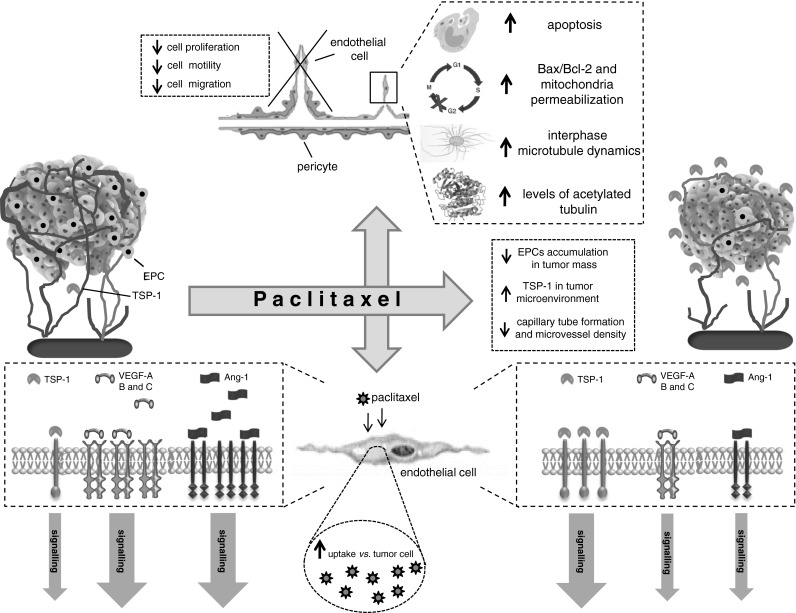
Molecular and cellular mechanisms of antiangiogenic activity of paclitaxel

The main molecular mechanisms for the antiangiogenic activity of paclitaxel are graphically summarized in Fig. 2. Pasquier and colleagues characterized two distinct effects of paclitaxel on human endothelial cell proliferation: a cytostatic effect at low paclitaxel concentrations, and a cytotoxic effect at higher concentrations [40]. The cytotoxic effect involved primarily those signaling networks that are reported to be impaired in tumor cells (i.e., microtubule network disturbance, G_2_-M arrest, increase in the Bax/Bcl-2 ratio, and mitochondria permeabilization) that result in apoptosis. Conversely, the cytostatic effect of paclitaxel involves the inhibition of endothelial cell proliferation without the induction apoptosis and without any structural modification to the microtubule network. In addition, low paclitaxel concentrations just initiate the apoptotic signaling pathway that is stopped upstream of mitochondria permeabilization and it does not lead to endothelial cell death [40]. Moreover, paclitaxel inhibits angiogenesis by an increase in microtubule dynamics in endothelial cells and by the impairment of interphase microtubule functions [41]. Other studies have uncovered additional mechanisms involved in the antiangiogenic effects of taxanes, including the degradation of heat shock protein 90 [42] and the inhibition of Rac1 and of Cdc42 activity [43]. It should be noted that these data have been obtained with docetaxel, but similar or analogous effects have yet to be reported with paclitaxel. Although the two drugs are very similar and belong to the same antineoplastic class of compounds, no data are available to show that such mechanisms could be responsible for the antiangiogenic activity of paclitaxel.

Recently, Bonezzi et al. [44] found that paclitaxel increases the levels of acetylated tubulin in HUVECs. The induction of tubulin hyperacetylation was concomitant with the inhibition of cell motility but not of cell proliferation, which only occurred at concentrations much higher than those that inhibited cell motility. In fact, the overexpression of a tubulin deacetylase such as the NAD-dependent histone deacetylase sirtuin-2 (SIRT2), increases cell motility and reduces cell response to the anti-motility activity of paclitaxel [44]. In the same article, Bonezzi et al. [44] found another possible target to explain the anti-motility activity of paclitaxel: forkhead box O3a (FOXO3a), a member of the family of the forkhead transcription factor FOXO, that negatively regulate cell motility. Indeed, the authors found that paclitaxel caused the translocation to the nucleus of the FOXO3a, which is a pre-requisite for its transcriptional activity.

In 2003 we [45] reported that protracted exposure of endothelial cells in vitro (144 h) to low concentrations of several different anticancer agents, including paclitaxel, caused overexpression of thrombospondin-1 (TSP-1), a potent and endothelial-specific inhibitor of angiogenesis. Elevated circulating TSP-1 levels were also detected in the plasma of mice treated with metronomic chemotherapy and, above all, the antiangiogenic and antitumor effects of low-dose continuous cyclophosphamide were absent in TSP-1-null C57BL/6 mice. These results suggested that TSP-1 acts as a mediator of the antiangiogenic effects of low-dose metronomic chemotherapy [45]. These data were later confirmed in other in vivo models. Indeed, systemic low-dose continuous treatment of a rat malignant prostate cancer model (Dunning AT-1) with paclitaxel induced the expression of TSP-1 in the tumor tissue, and inhibited tumor growth [46]. Moreover, metronomic paclitaxel chemotherapy also caused a dramatic increase in the expression of TSP-1 (whereas MTD chemotherapy did not) in a HT-29 colon cancer model [29] and in the highly metastatic 4T1 mouse model of breast cancer [30]. These findings support the hypothesis that the anti-tumour effect of low-dose metronomic paclitaxel is in part mediated by the induction of TSP-1 (Fig. 2).

Other interesting possible mechanisms include the downregulation of survival factors for endothelial cells such as Angiopoietin-1 (Ang-1) and VEGF-A. Ang-1 and angiopoietin-2 (Ang-2) are major ligands for the endothelium-specific tyrosine kinase receptor Tie-2, and they are important regulators of endothelial cell survival [46]. Long-term exposure (168 h) to low concentrations (2 nM) of paclitaxel significantly decreased Ang-1 and VEGF-A gene and protein expression in human ovarian cancer cell lines [47]. The downregulation of VEGF-A after paclitaxel treatment was also observed in tissue samples from 39 patients (diagnosed with invasive carcinoma) that were cultured with paclitaxel for 24 h. The mean VEGF-A levels were significantly lower in the treated samples compared to controls [48]. Moreover, the downregulation of VEGF-B and -D was also reported in response to paclitaxel treatment in ovarian carcinoma xenografts, whereas the expressions of Tie-1 and Tie-2 were significantly upregulated. In the same study, paclitaxel treatment significantly decreased the expression of VEGF-A, and it increased the expression of VEGFR-2 [49] (Fig. 2).

## New pharmaceutical formulations of paclitaxel enhance its antiangiogenic activity

In the last decade numerous efforts have focused on removing the use of Cremophor EL to decrease the hypersensitive side effects, or to improve the pharmacokinetic behavior, of paclitaxel. There are a variety of nanoparticle carrier systems being explored for chemotherapeutic drugs, including paclitaxel, such as liposomes, pegylated liposomes, protein nanoparticles, and polymeric nanoparticles [50].

Liposomes represent an established drug delivery system for lipophilic substances, and they act by improving the pharmacokinetics and the therapeutic index of the anticancer drugs [51]. Indeed, liposomal paclitaxel showed improved solubility and similar in vitro cytotoxicity against a variety of tumor cell lines compared to that of the Cremophor EL paclitaxel preparation [52]. However, a major limit in the clinical use of conventional liposome is its rapid clearance by the reticuloendothelial system after systemic administration, and is low bioavailability [53].

In contrast to the conventional liposomes, positively charged cationic liposomes have been developed to be preferentially bound to, and internalized by angiogenic and negatively charged, endothelial cells that are found in tumours and in areas of chronic inflammation [54]. Indeed, Thurston et al. [54] have shown that actively growing angiogenic endothelial cells exhibit a preferential uptake of cationic liposomes by endocytosis.

In 2003, Schmitt-Sody et al. [55] evaluated a novel neovascular targeting therapy, consisting of paclitaxel encapsulated in cationic liposomes, on tumor selectivity and antitumoral efficacy in comparison with the paclitaxel free drug in vivo (Table 2). The endothelial deposition of paclitaxel in tumor vessels significantly increased during the infusion time (compared to Cremophor EL paclitaxel preparation) and both the subcutaneous tumor growth and the appearance of regional lymph node metastases were significantly delayed in a amelanotic hamster melanoma model [55]. Around the same time, also Kunstfeld et al. [56] demonstrated that paclitaxel encapsulated in cationic liposomes, which have been shown to target blood vessels, prevented melanoma growth and invasive behavior, which overall resulted in an increase in the survival of the tumor-bearing mice. Moreover, liposome-encapsulated paclitaxel reduced vessel density at the interface between the tumor and the dermis, and reduced endothelial cell mitosis. In contrast, equimolar concentrations of paclitaxel solubilized in Cremophor EL produced only weak effects on tumor growth and did not reduce the mitotic index of the endothelium in vivo [56]. These data were also confirmed in an animal model of prostate cancer. Thus, paclitaxel encapsulated in cationic liposomes (EndoTAG-1) caused a significant decrease of microvessel density in tumors when compared to paclitaxel alone, confirming the relative suppression of angiogenesis when compared to the conventional treatment [57] (Table 2).

**Table 2:** Antiangiogenic effects of novel paclitaxel formulations in vitro and in vivo

Paclitaxel formulations	Models	Pharmacological effects	References
Paclitaxel encapsulated in cationic liposomes	In vivo amelanotic hamster melanoma A-Mel-3 model	Increased endothelial deposition of paclitaxel in tumor vessels; a remarkable retardation of tumor growth and appearance of regional lymph node metastases	[55]
Paclitaxel encapsulated in cationic liposomes	In vivo metastatic melanoma model	Inhibition of newly blood vessels, prevention of melanoma growth and invasiveness, improvement of mice survival	[56]
Paclitaxel encapsulated in cationic liposomes (EndoTAG-1)	In vivo prostate tumor xenograft	Decrease of microvessel density	[57]
Sterically stabilized liposomes containing paclitaxel (SSL-PTX)	HUVEC proliferation and migration in vitro; MDA-MB-231 breast cancer xenograft models	Inhibition of cell proliferation and migration at low concentrations; decrease of microvascular density of tumors treated with low doses of paclitaxel	[59]
Paclitaxel entrapped in emulsifying wax nanoparticles (PTX NPs)	Colon cancer xenograft models	Increase of antiangiogenic effect in the colon cancer xenograft models	[60]
Polymeric nanospheres loaded with paclitaxel	In vitro HUVEC culture and ex vivo rat aortic rings	Inhibition of proliferation and inhibition of endothelial sprouts	[61]
ABI-007, a cremophor EL-free, albumin-bound, 130-nm form of paclitaxel	Rat aortic rings, human endothelial cell proliferation and tube formation. Tumor xenografts	Inhibition of rat aortic microvessel outgrowth, human endothelial cell proliferation, and tube formation. Inhibition of tumor growth	[65]
Hyaluronic acid conjugates of paclitaxel (HA-PTX)	Female nude mice bearing ovarian cancer cells	Antitumor and antiangiogenic effects with a marked increase of TSP-1	[67]
PEG-VC-PABC-PTX; paclitaxel (PTX) conjugated with *p*-aminobenzylcarbonyl (PABC), valine-citrulline (VC), and polyethylene glycol (PEG)	MCF-7 tumor xenografts	Antitumor and antiangiogenic effects in vivo. Decrease of microvessel density	[68]

Another promising approach to the synthesis of liposomes with prolonged half-life is the grafting of the liposome with the inert and biocompatible polymer polyethylene glycol (PEG). This modification prevents the recognition of liposome by opsonins and therefore reduces its clearance by cells of the reticuloendothelial system [58]. The pegylated liposome is therefore often referred to as a sterically stabilized liposome (SSL). Huang et al. [59] evaluated the antiangiogenic activity of sterically stabilized liposomes containing paclitaxel (SSL-PTX). SSL-PTX effectively inhibited endothelial cell proliferation and migration in a concentration-dependent manner and its metronomic administration induced marked tumor growth inhibition in the MDA-MB-231 xenograft model via an antiangiogenic mechanism (i.e., reduced microvessel density), when compared to the injection of paclitaxel formulated in Cremophor EL [59].

In order to improve the activity of low concentrations of Cremophor EL-free paclitaxel on endothelial cell proliferation, motility, and tube formation, and to facilitate the delivery to blood vessels, new nanoparticles have been synthesized and tested in vitro and in vivo (Table 2). Colloidal carriers have been shown to improve tumor therapy by increased drug delivery to tumors resulting directly from the enhanced permeability and retention of the drug. Thus, paclitaxel entrapped in emulsifying wax nanoparticles (PTX NPs) showed enhanced efficacy against colon cancer xenograft models, overcoming paclitaxel resistance and increasing the drug’s antiangiogenic effect [60]. Moreover, the development of polymeric nanospheres (NPs), that are able to selectively target the activated vascular endothelium and to deliver co-encapsulated anti-angiogenic agents, has also been studied with paclitaxel [61]. Indeed, NPs loaded with paclitaxel have been successfully tested for their anti-angiogenic efficacy in HUVECs in vitro, and in rat aorta rings ex vivo. These effects were more potent than those observed with free paclitaxel [61].

Albumin is a very attractive drug carrier for antineoplastic drugs [62]. It has been demonstrated that albumin helps endothelial transcytosis of protein-bound and unbound plasma constituents, mainly via a cell-surface glycoprotein receptor (gp60). The gp60 protein binds to caveolin-1 (an intracellular protein) which results in the subsequent formation of transcytotic vesicles. In addition, gp60 also binds to osteonectin. Both caveolin-1 and osteonectin are frequently present in certain neoplasms (such as breast, lung, and prostate cancer), which could account for the accumulation of albumin in some tumors. Such accumulation of albumin could facilitate intratumoral accumulation of albumin-bound drugs [62]. In that respect, albumin-bound paclitaxel ABI-007 is a novel, cremophor EL-free, 130-nm particle formulation of paclitaxel [63]. This formulation is obtained by high-pressure homogenization of paclitaxel with serum albumin, resulting in a nanoparticle colloidal suspension [64]. Preclinical studies on human breast cancer xenografts have demonstrated that ABI-007 has a higher penetration into tumor cells, with increased antitumor activity, compared to an equal dose of standard paclitaxel [63]. Interestingly, the antiangiogenic activity of ABI-007 was described in the pivotal study by Ng et al. [65], who employed a therapeutic metronomic approach. Indeed, recent data showed that clinically relevant concentrations of vehicles such as Cremophor EL and polysorbate 80 nullify the antiangiogenic activity of taxanes [66]. These results could hamper the usefulness of paclitaxel metronomic regimens. In order to solve this problem, Ng et al. [65] tested ABI-007 both in in vitro and in in vivo models (Table 2). ABI-007 was found to significantly inhibit rat aortic microvessel outgrowth, human endothelial cell proliferation, and tube formation. Moreover, metronomic ABI-007 significantly suppressed tumor growth in xenograft models with a very low toxicity profile [65].

Recently, hyaluronic acid (HA) conjugates of paclitaxel (HA-PTX) were shown to produce strong anti-tumor effects when dosed metronomically. They also induced anti-angiogenic effects of greater magnitude than those achieved with MTD administration, or with free PTX administration in female nude mice bearing ovarian cancer cells [67]. Moreover, in a taxane-resistant model of ovarian cancer, a significant reduction in tumor weight was noted in the metronomic HA-PTX treated groups, whereas the response of the MTD group was not statistically significant. Metronomic HA-PTX treatment resulted in marked increases in TSP-1, confirming the anti-angiogenic mechanism of action of this novel formulation [67].

Another interesting new paclitaxel formulation was published by Liang et al. [68]. The novel paclitaxel conjugate (PEG-VC-PABC-PTX) was designed and synthesized using p-aminobenzylcarbonyl (PABC), a spacer, and valine-citrulline (VC), a substrate of cathepsin B, to link polyethylene glycol (PEG) and PTX. The PEG-VC-PABC-PTX showed significant antitumor and anti-angiogenic effects in vivo, while the control conjugate was almost ineffective [68].

The development of an oral formulation of paclitaxel is necessary for the clinical development of metronomic chemotherapy. Moes et al. [69] have increased the oral bioavailability of paclitaxel by combining ritonavir with a new oral solid dispersion formulation of paclitaxel (1/11 w/w paclitaxel, 9/11 w/w polyvinylpyrrolidone K30, and 1/11 w/w sodium lauryl sulfate)—which they termed ModraPac001. Currently, the ModraPac001 formulation is being applied in the first clinical trial with oral metronomic chemotherapy of paclitaxel [69].

Among all the new formulations of paclitaxel with demonstrated antiangiogenic characteristics, ABI-007 was the most successful in clinical practice. Indeed, it was initially approved by the US Food and drug Administration in January 2005, and indicated for use with pretreated metastatic breast cancer patients. ABI-007 is currently approved in 42 countries, including the European Union, Canada, India and Japan [64]. Thus, in a relatively short period, there has been an impressive improvement in our understanding of the antiangiogenic mechanism of paclitaxel. Further laboratory and clinical research is necessary to maximize the benefits of the antiangiogenic properties of paclitaxel for the treatment of patients with various cancers. In this context, the knowledge and investigation of metronomic chemotherapy based on the new formulations of paclitaxel, such as ABI-007, HA-PTX or ModraPac001, constitutes today one of the most exciting strategies for improving the clinical control of angiogenesis in primary and metastatic tumors [70]. On the other hand, such treatment strategies should be based on solid and significant results of randomized phase III clinical trials, and not only on their safety profiles or on their impact on the quality of life and on patient preference. Nonetheless, tolerability and compliance will probably become the most important factors in the future, according to the emerging overall importance of quality of life in cancer care [71]. The availability of new drugs for metronomic chemotherapeutic strategies (i.e. ABI-007, HA-PTX and ModraPac001), alone or in association with other targeted molecules, will give the oncologist many different effective treatment options for metastatic patients that are already resistant to standard doses of chemotherapy [70]. Indeed, both preclinical [72] and clinical studies [73, 74] have suggested that metronomic chemotherapy could be effective in stabilizing tumors already resistant to the same drug MTD treatments. Moreover, the ability of paclitaxel to inhibit different pathways of the angiogenic process may be a great advantage in the treatment of tumors already resistant to antiangiogenic drugs with a specific target (e.g. bevacizumab or sunitinib).

## Conclusion

The aim of this review was to summarize the published data on the molecular and pharmacological bases of the antiangiogenic activity of paclitaxel, and to underline this particular pharmacodynamic characteristic of the drug for possible future clinical development. In fact, paclitaxel (and its novel formulations) both at MTD and at low dose schedules demonstrated significant antiangiogenic activity. This effect could be ascribed both to a cytotoxic or to a cytostatic activity towards activated endothelial cells, or to the inhibition of capillary formation and cell migration. Moreover, paclitaxel has been shown to inhibit the release of VEGF and Ang-1 by tumor cells, and to increase the secretion of TSP-1 to the tumor microenvironment. It is therefore possible that the different antiangiogenic mechanisms of paclitaxel described in this review may explain the antiangiogenic activity of this drug in different human tumors, such as prostate and breast cancer. Indeed, the angiogenic process in these tumors may depend on the balance of different proangiogenic factors and endogenous angiogenic inhibitors. Paclitaxel can definitely affect those different pathways, resulting in an overall clinical activity, and one that is independent from the direct cytotoxic effects of the drug on cancer cells. Thus, based on the available published preclinical data, paclitaxel (and especially its novel formulations) may permit novel and rational therapeutic strategies to control tumor progression in patients.
